# Supplementary figures and images for: Biomarkers and Data Visualization of Insulin Resistance and Metabolic Syndrome: An Applicable Approach
Source: Life (Basel). 2024 Sep 21;14(9):1197. doi: 10.3390/life14091197 (PMC11433343; doi:10.3390/life14091197)

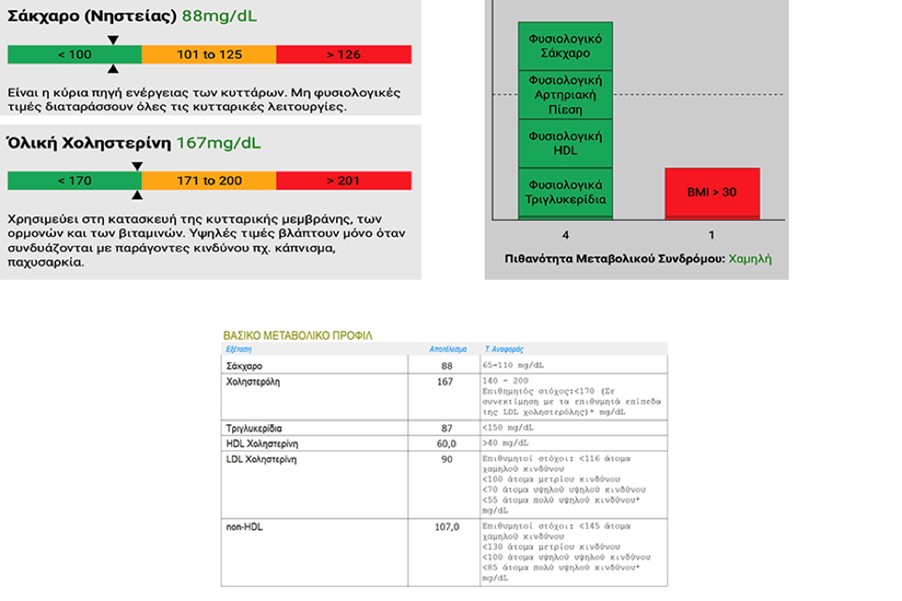

Supplement: Supplementary file 1 [file life-14-01197-s001.zip › life-3172786-supplementary.jpg]
